# Association between sunlight exposure and risk of age-related macular degeneration: a meta-analysis

**DOI:** 10.1186/s12886-018-1004-y

**Published:** 2018-12-20

**Authors:** Hongjie Zhou, Huina Zhang, Aiqing Yu, Jiajun Xie

**Affiliations:** 1 Hangzhou Hospital for the Prevention and Treatment of Occupational Diseases, Zhejiang, Hangzhou China; 2 Department of Ophthalmology, College of Medicine, the 2nd Affiliated Hospital of Zhejiang University, Zhejiang, Hangzhou China

**Keywords:** Sunlight, Macular degeneration, Meta-analysis, Risk factor

## Abstract

**Background:**

A substantial number of epidemiological studies have investigated the possible associations between sunlight exposure and Age-related Macular Degeneration (AMD), but the results from studies are inconsistent. The aim of this meta-analysis was to evaluate the association between sunlight exposure and the risk of AMD.

**Methods:**

Relevant studies were searched using databases including PubMed, EMBASE, and Web of Science database. Two authors independently extracted data and assessed study quality. The random-effects model was used to calculate the pooled covariates-adjusted odds ratio (OR). Subgroup analyses based on study design, stage of AMD, method of exposure assessment, and study latitude were carried out. The heterogeneity across the studies was tested, as was publication bias.

**Results:**

Fourteen eligible studies including 43,934 individuals based on the inclusion criteria were analyzed. The pooled OR for sunlight exposure and AMD was 1.10 (95% CI = 0.98–1.23). In addition, similar insignificant results were observed in further subgroup analyses based on stage of AMD, method of exposure assessment, and study latitude. Sun-avoidance behavior did not decrease the risk of AMD (OR = 1.12, 95% CI = 0.76–1.67). Moderate heterogeneity was observed in most of analyses.

**Conclusion:**

The results indicate that sunlight exposure may not be associated with increased risk of AMD based on current published data.

## Background

Age-related macular degeneration (AMD) is a progressive chronic disease of the central retina and a leading cause of vision loss worldwide [1]. It accounts for approximately 50% of all cases of central blindness among older people in western countries [2]. Annual incidence of late AMD in Americans aged ≥50 years was 3.5 per 1000 (95% CI = 2.5–4.7 per 1000), incidence rates approximately quadrupled per decade in age [3].

Generally, AMD is divided into early AMD and late AMD according to the international classification and grading system [4]. Early AMD is frequently described as age-related maculopathy (ARM). Late AMD can be further classified into geographic atrophy (“dry”) and neovascular (“wet”) types. The geographic atrophy type accounts for about 80% of all late AMD cases for few available treatment [5].

The causes of AMD are still not well understood. Numerous studies have been performed to identify risk factors for AMD. Many risk factors have been identified, including aging, gender, genetics, alcohol consumption, smoking, diet, and cardiovascular function [6, 7]. Several studies have investigated the possible associations between sunlight exposure and AMD; however, the results of those studies are inconsistent [6]. The aim of this study was to comprehensively evaluate the association between sunlight exposure and the risk of AMD by performing a meta-analysis of all relevant studies.

## Methods

### Search strategy

This meta-analysis was conducted and reported according to the meta-analysis of observational studies in epidemiology guidelines [8]. We conducted a literature search in three databases: PubMed, EMBASE, and Web of Science databases (up to 31 December 2017) by using the keywords “sunlight, light, ultraviolet, or risk factor” and “macular degeneration, maculopathy, AMD”. Furthermore, manual search of the references from original studies or relevant reviews were performed for any other pertinent studies. The language was restricted to English, and the search included all studies conducted on epidemiological investigation. Full texts or abstracts of all related literature were then reviewed. Two investigators retrieved the literature independently.

### Inclusion and exclusion criteria

The included studies were required to meet all of the following criteria: (1) the studies should refer to the association between AMD and sunlight exposure; (2) calculable information and effects estimates like odds ratio (OR), hazard ratio with 95% confidence interval (CI), or *P*-value were provided; and (3) “visible light exposure” and “blue light exposure” were regarded as sunlight exposure. Exclusion criteria were: (1) duplicate subjects; (2) abstracts, case reports, comments, reviews and experimental study designs in laboratory settings; (3) other languages; and (4) studies with no available data.

The Newcastle-Ottawa Scale (NOS) [9] was used to evaluate the quality of each observational study. According to the NOS score standard, all studies could be classified as low-quality (scores of 0–4), moderate-quality (scores of 5–6) and high-quality (scores≥7). We excluded studies from the meta-analysis if they were of low-quality.

Each included study must be an unrelated study. When subjects overlapped between two studies, only the one with larger sample size was selected, while another with smaller sample size was excluded.

### Data extraction

Data extraction was performed by two independent investigators from the included studies. Any conflicting were resolved by discussion with each other. The following information was extracted: first author, year of publication, study design, study populations, study location, the definition and classification of AMD, exposure assessment criteria, confounding variables, and main results. If articles reported the OR and its *P*-value but not the 95% CI, it was calculated for our study. If the articles only provided the results for early or late AMD with sunlight exposure, but not for all categories of AMD, we calculated the pooled results. Assessment criteria for exposure to sunlight included leisure time in the sun, work time outside, calculated doses of sunlight, and living in a sunny area or climate. Various criteria were used to classify the exposed and unexposed groups: living in a sunny climate/region, leisure time outdoors, work time outdoors, average doses of light, and avoidance of the sun. Google Earth was used to identify the latitude of each study location, and we regard < 40° as lower latitude and ≥ 40° as higher latitude. When additional information was needed, we contacted corresponding authors.

Whether the International Classification and Grading system (ICGS) or the Wisconsin Age-Related Maculopathy Grading System (WARMGS) was used was recorded [4, 10]. Early AMD was diagnosed based on the presence of soft drusen or pigmentary abnormalities in the absence of signs of late-AMD. Meanwhile, late AMD was diagnosed based on the presence of neovascular AMD or central geographic atrophy.

### Statistical analysis

The χ^2^-based Q-tests and inconsistency score (I^2^) were used to assess the heterogeneity among studies [11]. Significant heterogeneity was detected if the χ^2^ test was significant (*P* < 0.05). The high, moderate, low and no heterogeneity was considered corresponding to the I^2^ value of ≥75%, 50–74%, 25–49%, < 25%, respectively [11]. Then the random and the fixed effect model were performed According to the results of heterogeneity test. OR and 95% CI were calculated to assess the risk of AMD from exposure to sunlight. Subgroup analyses were performed to estimate the accuracy and stability of the pooled effect size, based on study design, the grade of AMD, or the method of exposure assessment. Potential publication bias was assessed by Begg’s funnel plots [12] and Egger’s test [13]. Asymmetry of the funnel plots was checked for the existence of publication bias by examining the distribution of the effect size of the OR. Egger’s test was performed to assess the degree of asymmetry; *P* < 0.05 was considered a statistically significant publication bias. All analyses were conducted using STATA software (version 11.0; StataCorp LP, College Station, TX, USA) [13], and the statistically significant level is 0.05.

## Results

### Literature search

**Fig. 1:**
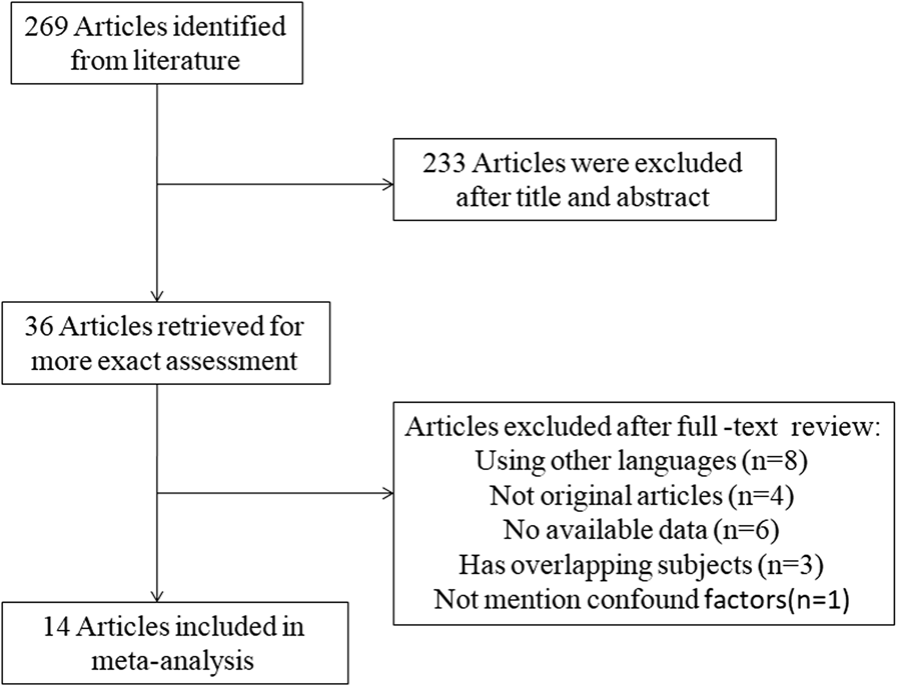
Flow diagram of studies identification

A flow diagram of our literature search is shown in Fig. 1. After screening titles and abstracts (including conference abstracts), 233 articles were excluded from an initial 269 potentially relevant articles. After full-text review of the remaining 36 articles, 22 articles were excluded for the following reasons: 8 articles were not in English, 4 articles were review or meta-analysis but not original articles, 6 articles failed to report the available data, 3 articles had overlapping subjects with others, and 1 article failed to mention confounding factors. Finally, 14 studies that met the inclusion criteria were identified as eligible (Fig. 1).

### Study characteristics

A total of 14 studies with 43,934 individuals were included in this meta-analysis, as detailed in Table 1. Among the 14, 7 were case control studies [14–20], 6 were cross-sectional studies [21–26] and 1 was a cohort study [27]. A total of 8 studies assessed sunlight exposure levels based on time spent outdoors [14, 18, 20–22, 24, 26, 27], 5 studies were based on occupational exposure to sunlight [14, 19, 20, 25, 27], 2 studies were based on living in a sunny region or sunny climate [16, 20], 3 studies were based on calculated doses of sunlight or blue light [15, 23, 25], and 6 studies were based on avoidance of the sun. [16, 17, 23–25, 27]

**Table 1:** Characteristics of epidemiological studies of the association between sunlight exposure and AMD risk

First author, year	Study population (location)	Design	Latitude	Definition of AMD	Covariates	Exposure assessment, criteria	Main results^a^		
							ALL	Early AMD	Late AMD
Eye disease case–control study group, (1992)	421 cases and 615 controls (USA)	Case–control	42	Described in paper	age, sex, and clinic	summer Leisure time in the sun	1.1 (0.7–1.7)	–	–
						occupational outdoors	1.1 (0.6–2.1)		
						wearing sunglasses	0.9 (0.5–1.5)		
Taylor, (1992)	838 watermen (USA)	Cross-sectional	37	Described in paper	age	average doses of blue light	Pooled	Pooled	
							1.045 (0.977–1.117)	1.031 (0.969–1.099)	1.36 (1–1.85)
Delcourt, (2001)	2584 residents (France)	Cross-sectional	43.4	ICGS	age and sex	average doses of sunlight	Pooled		
						occupational outdoors	0.704 (0.529–0.939)	0.73 (0.54–0.98)	0.44 (0.15–1.31)
						wearing sunglasses	0.996 (0.748–1.325)	1.02 (0.76–1.38)	0.76 (0.28–2.07)
							0.818 (0.606–1.104)	0.87 (0.71–1.07)	0.56 (0.26–1.19)
Tomany, (2004)	Cohort of 6448 persons (USA)	cohort	43	WARMGS	age, sex, smoking, iris color, et al	leisure time outdoors in summer	Pooled		
						occupational outdoors	1.832 (0.934–3.591)	2.20 (1.02–4.73)	0.99 (0.24–3.99)
						wearing hats and sunglasses	1.163 (0.851–1.589)	1.18 (0.85–1.64)	1.02 (0.38–2.74)
							1.076 (0.837–1.383)	1.09 (0.83–1.44)	1.01 (0.55–1.86)
Bai, (2005)	2835 people (China)	Cross-sectional	35	ICGS	age, sex, smoking, drinking, et al	time outdoors	0.52 (0.23–1.17)	–	–
						using adumbral tools	0.68 (0.23–0.80)		
Khan, (2006)	446 cases and 283 controls (Britain)	Case–control	53	ICGS	age, sex, and smoking	Sunny climate	1.18 (0.74–1.89)	–	–
						avoidance measures undertaken	1.0 (0.58–1.72)		
Fletcher, (2008)	2283 cases and 2117 controls (Europe)	Case–control	50	ICGS	age and sex	average doses of blue light	1.09 (0.84–1.41)	–	–
Butt, (2011)	1019 participants (USA)	Cross sectional	35	WARMGS	age, sex	time outdoors	1.03 (1.01, 1.04)	–	–
Nano, (2013)	175 cases and 175 controls (Argentina)	Case–control	34	Not mention	age, race, smoking, et al	time outdoors	3.281 (1.91–5.62)	–	–
Ristau, (2014)	445 cases and 1014 controls (Cologne Germany)	Case–control	51	Not mention	age, alcohol use, allergy, education, et al	occupational outdoors	–	–	2.02 (1.04–3.91) “CI” was calculated
Park, (2014)	14,352 participants (Korean)	Cross-sectional	34–38	ICGS	age, sex, and smoking	time outdoors	Pooled		
							1.057 (0.885–1.262)	1.07 (0.89–1.28)	0.83 (0.37–1.86)
Huang, (2014)	3000 residents (Taiwan)	Cross-sectional	23	WARMGS	age, sex, smoking, drinking, et al	time outdoors	Pooled		
							1.768 (0.492–6.351)	0.947 (0.578–1.551)	3.497 (1.683–7.268)
Schick, (2016)	1931 cases and 1770 controls (Europe)	Case–control	50	Described in paper	age, sex, and smoking	time outdoors	Pooled		
						occupational outdoors	3.334 (1.563–7.109)	5.54 (1.14–21.34)	2.77 (1.25–6.16)
							1.765 (0.837–3.722)	1.20 (0.85–1.71)	2.57 (1.89–3.48)
Lazreg, (2016)	1183 cases and 827 controls (Algeria)	Case–control	36	ICGS	age and sex	sunny area	Pooled		
						Usual solar protection	0.596 (0.257–1.384)	0.86 (0.45–1.66)	0.36 (0.14–0.92)
							5.581 (2.269–12.72)	4.93 (1.94–12.51)	8.63 (2.12–35.10)

^a^Data are presented as odds ratio (95% confidence interval)

Assessments and definitions of AMD varied among the studies. Standardized criteria for diagnosis of AMD were used in some studies [15–17, 21, 22, 24–27], while in others, cases were diagnosed medically by an ophthalmologist or medical record review to identify the case [14, 18–20, 23]. Two stages of AMD (early and late) were analyzed separately in 7 studies [17, 20–23, 25, 27].

### Quantitative synthesis

**Fig. 2:**
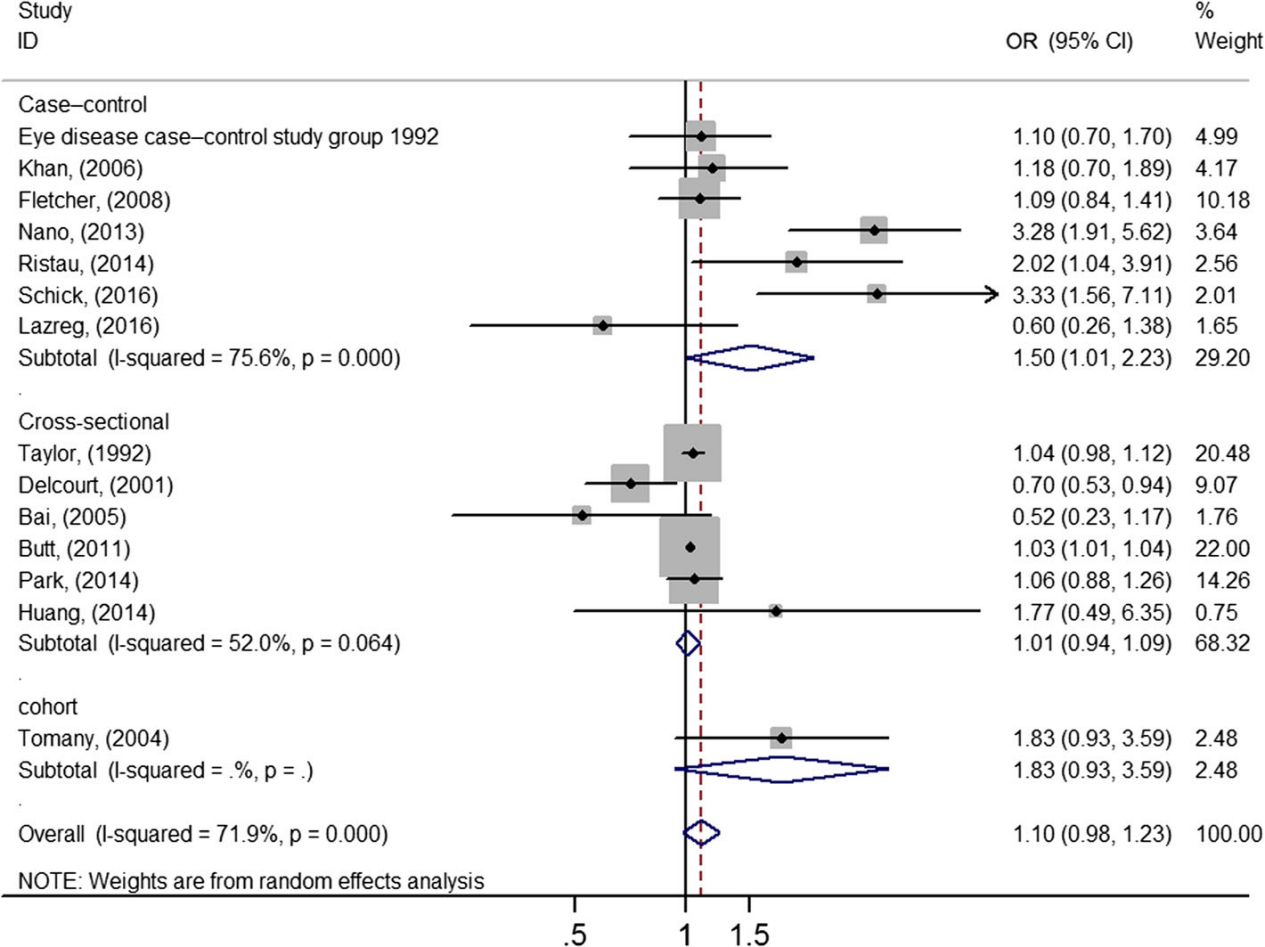
Forest plot of the association between sunlight exposure and risk of AMD. It shows a pooled OR of 1.10 (95% CI = 0.98–1.23, *P* = 0.093), demonstrated that there was no relationship between AMD and sunlight exposure. Abbreviations: OR, odds ratio; CI, confidence interval

The main results of this meta-analysis and the heterogeneity test are shown in Table 2 and Fig. 2. The data demonstrated that there was no relationship between AMD and sunlight exposure (OR = 1.10, 95% CI = 0.98–1.23).

**Table 2:** Subgroup analysis of sunlight exposure and the risk of AMD

Subgroup analysis	All AMD			Early stage			Late stage		
	No.^a^	OR(95% CI)	I^2^ (*P*) ^b^	No. ^a^	OR(95% CI)	I^2^ (*P*) ^b^	No. ^a^	OR(95% CI)	I^2^ (*P*) ^b^
pooled	14	1.10 (0.98–1.23)	71.9% (0.000)	7	1.02 (0.85–1.22)	58.7% (0.024)	7	1.16 (0.66–2.04)	73.8% (0.001)
Study design									
Case–control	7	1.50 (1.01–2.23)	75.6% (0.000)	2	1.94 (0.32–11.83)	80.7% (0.023)	2	1.01 (0.14–7.49)	90.5% (0.001)
Cross-sectional	6	1.01 (0.94–1.09)	52.0% (0.064)	4	0.99 (0.87–1.12)	43.5% (0.151)	4	1.24 (0.63–2.46)	75.0% (0.007)
Exposure assessment method									
Time work outdoors	5	1.16 (0.97–1.40)	20.5% (0.284)	3	1.12 (0.93–1.35)	0% (0.732)	4	1.65 (0.96–2.84)	61.0% (0.053)
Quantitative data	3	0.95 (0.77–1.19)	71.9% (0.029)	2	0.90 (0.64–1.25)	79.6% (0.027)	2	0.88 (0.30–2.58)	74.1% (0.050)
Using avoidance	6	1.12 (0.76–1.67)	78.7% (0.000)	3	1.31 (0.78–2.21)	85.1% (0.001)	3	1.44 (0.44–4.69)	82.3% (0.004)
Study latitude									
lower latitude	7	1.07 (0.95–1.20)	73.9% (0.001)	4	1.03 (0.97–1.09)	0% (0.903)	4	1.15 (0.55–2.43)	86.6% (0.001)
higher latitude	7	1.30 (0.93–1.81)	73.9% (0.001)	3	1.76 (0.58–5.33)	84.6% (0.001)	3	1.12 (0.34–3.70)	73.0% (0.024)

^a^ Number of studies

^b^ Percentage of total variation across studies attributable to statistical heterogeneity rather than to chance (25%, low; 50%, moderate; 75%, high); *P*-value for heterogeneity test

In subgroup analyses, an increased risk of AMD was found in case–control studies (OR = 1.5, 95% CI = 1.01–2.23), but not in cross-sectional studies (OR = 1.01, 95% CI = 0.94–1.09). In subgroups classified by the stage of AMD, no significant outcome was found for early AMD (OR = 1.02, 95% CI = 0.85–1.22) or late AMD (OR = 1.16, 95% CI = 0.66–2.04). In addition, no significant relationship was found in a subgroup analysis classified by exposure assessment methods: time worked outdoors (OR = 1.16, 95% CI = 0.97–1.40), quantitative data (OR = 0.95, 95% CI = 0.77–1.19), or sun-avoidance behavior (OR = 1.12, 95% CI = 0.76–1.67). Neither lower latitude (OR = 1.14, 95% CI = 0.85–1.53), nor higher latitude (OR 1.3, 95% CI = 0.93–1.81) affected the study results, which suggests that latitude doesn’t affect the strength of the association between sunlight exposure and AMD. Moderate heterogeneity was present in most of analyses.

### Diagnosis of publication biases

**Fig. 3:**
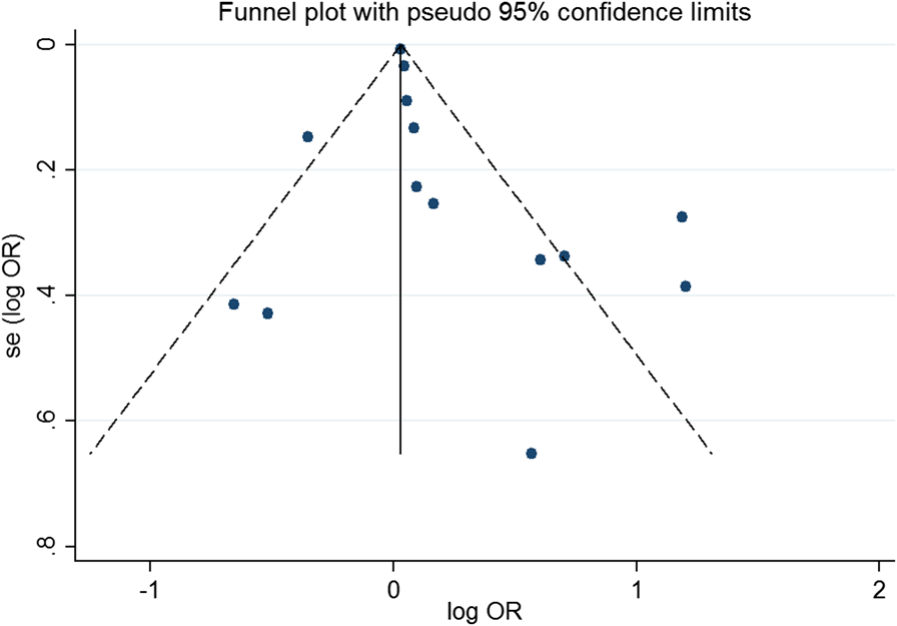
Funnel plot analysis to detect publication bias. Egger’s test suggested no statistically significant asymmetry of the funnel plot (*P* = 0.25), indicating no evidence of substantial publication bias

Begg’s funnel plot and the Egger-weighted regression were applied and no significant publication bias was detected in our meta-analysis, as shown in Fig. 3.

## Discussion

Our meta-analysis showed that sunlight exposure was not associated with an increased risk of AMD in a pooled analysis. In subgroup analyses, only one mildly significant association was found in the case–control studies, but not in the cross-sectional studies. No significant association was observed in the stratified analyses by degree of AMD, exposure assessment method, and study latitude.

Another meta-analysis study for sunlight and AMD was conducted by Sui five years ago [28]: Fourteen studies were pooled, and a statistically significant association between sunlight and the risk of AMD (OR = 1.379, 95% CI = 1.091–1.745) was found, which is contrary to the conclusion reached by us. However, several limitations of that study need to be considered. Firstly, new studies published in the last five years were not included in that study. Secondly, due to different strategies regarding selection criteria, six studies included in Sui’s study were excluded in our study: three articles were MD or phD thesis in chinese, two articles failed to adjust for confounders [29, 30], and one articles had overlapping subjects with others [31]. Lastly, a misclassification of the study design was found, in which a case–control study was mistakenly referred to as a cross-sectional study [15].

Many in-vitro and in-vivo studies have been focused on the association of sunlight and retinal pigment epithelium (RPE) cells [32]. It has been unequivocally demonstrated that either UV or blue light would result in damage, leading to a decline in the vitality of RPE cells [33–36]. On the contrary, epidemiological evidence of the association between sunlight exposure and AMD is mixed, with our meta-analysis showing no relationship. The possible reason is that most light is absorbed and blocked by ocular media (cornea, lens, and vitreous), and only very small amounts can reach the retina [37]. Besides, the retina possesses inherent protection against damage via antioxidant enzymes such as superoxidase dismutase (SOD), catalase, and glutathione peroxidase; macular pigments such as melanin, hemoglobin, and flavoproteins, which absorb light; and the fact that photoreceptor cells can shed potentially damaged outer segment discs [6].

Methods of assessing exposure were critical for evaluating the quality of the articles included in the study. However, it was difficult to quantify sunlight exposure objectively. Self-reported data of time outdoors are easy to obtain but imprecise and could be influenced by many factors, such as latitude, climate, and sun-avoidance behaviors. In our analysis, most of the articles used time spent outdoors to assess sunlight exposure. Only three of fourteen articles had calculated the average dose of sunlight for each participant after analyzing these factors but still found no association between exposure to sunlight and AMD (OR = 0.95, 95% CI = 0.77–1.19). In addition, sun-avoidance behaviors were analyzed, showing that subjects who used sunglasses or hats regularly didn’t have a decreased risk of AMD.

We also analyzed the relationship between sunlight and AMD based on low and high latitude, and all of the results turned out to be statistically insignificant. Residents living in low latitudes suffer more solar insolation than in high latitudes, but a smaller OR value was indicated in low latitudes, which didn’t support our hypothesis about sunlight and AMD. Conversely, a systematic literature review was conducted to evaluate geographic variability in the prevalence rates of AMD, and proximity to the equator and higher solar insolation were both found to be significantly associated with a lower prevalence of AMD [38]. This finding may support the hypothesis of the role of vitamin D in the pathogenesis of AMD. Many studies indicated a significant correlation between reduced plasma levels of vitamin D and a higher prevalence of the disease [39–41]. As exposure to UV radiation is the most important source of Vitamin D synthesis, residents in lower latitudes surely have a higher vitamin D status to help resist the disease [41].

On the other hand, all of these assessments of total sunlight exposure are based on questionnaires, and the accuracy of the data obtained depends heavily on question quality and respondents’ memory. To reduce these biases, some studies used sunlight-related factors such as iris, skin, or hair color, and sun sensitivity to assess exposure to sunlight [7]. 10 years of longitudinal data from the Blue Mountains did not find a consistent pattern of association between sunlight-related factors and incidence of AMD [38]; The Beaver Dam Eye Study reported that iris color was inconsistently related to the 10-year incidence of early ARM and to ARM progression, but no associations were found between late ARM and iris color, hair color, and skin sun sensitivity [8]. Hirakawa used other proxy measures, demonstrating that significantly more facial wrinkling and less facial hyperpigmentation was present in late ARM cases [42]. Overall, the relationship between proxy measures for sunlight exposure and AMD was not conclusive. However, the common limitation in those studies was that the correlation between such proxies and true sunlight exposure is unknown.

The limitations of this meta-analysis should be noted as well. First, the eligible studies only covered those that were written in English, so there may have been a language bias. Second, the latency period of AMD is long [43], an information bias is inevitable in the included studies for the time lag between sunlight exposure and manifestation of AMD. Third, the criteria for AMD diagnosis was varied across studies: 5 were based on WARMGS, 6 were based on ICGS, 3 were described in the paper, and 2 were not mentioned. Lastly, different methods were applied to estimate the exposure level among studies, so there was potential for a confounding effect in the pooled analysis for all studies.

## Conclusions

This meta-analysis included fourteen studies on the association between AMD risk and sunlight. Although there are potential limitations in studies, we did a rigorous analysis via stratification based on study design, stage of AMD, method of exposure assessment, and study latitude to reduce the bias. The results indicate that sunlight exposure may not be associated with an increased risk of AMD based on current published data.

## Abbreviations

AMD: Age-related macular degeneration
ARM: Age-related maculopathy
CI: Confidence interval
ICGS: International Classification and Grading system
NOS: The Newcastle-Ottawa Scale
OR: Odds ratio
WARMGS: Wisconsin Age-Related Maculopathy Grading System
